# Probability of achieving bone mineral density treatment targets with abaloparatide and teriparatide

**DOI:** 10.1093/jbmr/zjaf053

**Published:** 2025-04-11

**Authors:** Felicia Cosman, Bruce H Mitlak, Yamei Wang, Leny Pearman, Carolina A Moreira, E Michael Lewiecki, Steven R Cummings

**Affiliations:** Department of Medicine, Columbia University College of Physicians and Surgeons, New York, NY 10032, United States; Clinical Development, Radius Health Inc., Boston, MA 02210, United States; Biostatistics, Radius Health Inc., Boston, MA 02210, United States; Field Medical, Radius Health Inc, Boston, MA 02210, United States; Endocrine Division (SEMPR), Federal University of Parana, Curitiba, PR 80060-000, Brazil; Division of Metabolic Bone Diseases, University of New Mexico Health Sciences Center and New Mexico Clinical Research & Osteoporosis Center, Albuquerque, NM 87106, United States; San Francisco Coordinating Center, California Pacific Medical Center Research Institute, San Francisco, CA 94158, United States; Department of Epidemiology and Biostatistics, University of California, San Francisco, CA 94158, United States

**Keywords:** anabolic, antiresorptive, fracture prevention, fracture risk assessment, osteoporosis

## Abstract

The goal of treatment for women at high risk of fracture who have a T-score ≤−2.5 is to mitigate fracture risk by achieving T-scores at least above −2.5. In the ACTIVE trial, 2463 women with osteoporosis aged 49-86 yr were treated for 18 mo with abaloparatide (80 μg), teriparatide (20 μg), or placebo. In ACTIVExtend, eligible women from the abaloparatide and placebo groups received weekly treatment with 70 mg of alendronate for 2 additional years. This post hoc analysis of ACTIVE and ACTIVExtend included women with baseline TH or LS T-scores ≤−2.5. Logistic regression was used to predict the probability of achieving a T-score >−2.5 at the TH or LS during 18 mo of treatment with abaloparatide or teriparatide and during 3.5 yr with the sequence of abaloparatide/alendronate compared to placebo/alendronate. At baseline, 23% and 74% of women enrolled in ACTIVE had T-scores ≤−2.5 at the TH and LS, respectively. Over 18 mo of treatment, more than 50% of women were likely to achieve TH T-scores >−2.5, with baseline TH T-scores as low as −2.7 for both abaloparatide and teriparatide. More than 50% of women were predicted to achieve an LS T-score >−2.5 with a baseline LS T-score as low as −3.3 for abaloparatide or −3.2 on teriparatide. Over 3.5 yr of sequential treatment with abaloparatide/alendronate, >50% of women with baseline TH T-scores ≥−2.9 and LS T-scores ≥−3.5 were predicted to achieve T-scores >−2.5, respectively. A patient’s BMD at baseline and the probability of achieving target T-scores with treatment should be considered when determining that treatment should be initiated in patients at high or very high risk of fracture.

## Graphical Abstract

**Graphical Abstract:**
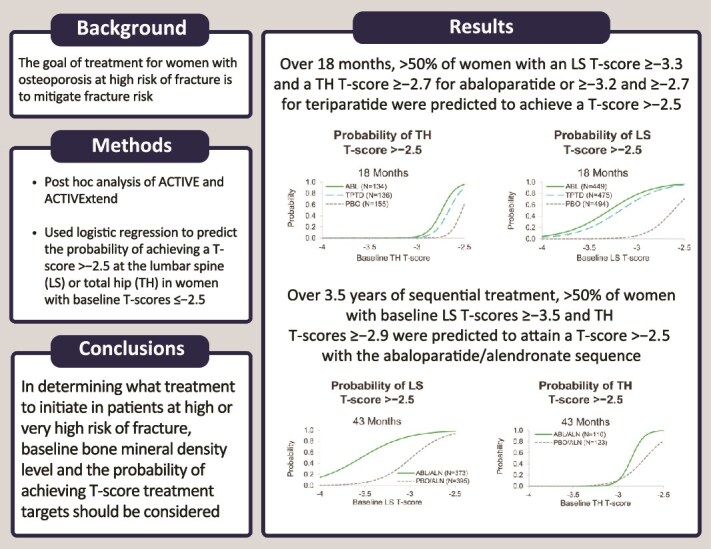

## Introduction

The goal of treatment for women with osteoporosis is to reduce the risk of fractures.^1^ However, fractures occur at relatively low rates, requiring large clinical trials of long duration to assess.^2^ Increases in BMD with osteoporosis treatment (vs placebo) have been associated with fracture risk reduction in a large meta-analysis of 111 000 patients participating in 38 randomized, placebo-controlled trials of 19 different therapies for osteoporosis.^2^ These data support the use of BMD change with treatment vs placebo as a surrogate endpoint for fracture risk in clinical trials. Furthermore, BMD levels in the TH and/or FN attained on treatment with multiple osteoporosis medications, including alendronate, zoledronic acid, denosumab, and romosozumab, are associated with the subsequent risk of fracture.^3–6^ After osteoporosis therapy, women who have T-scores which remain below −2.5 have significantly higher fracture risk than those who attain T-scores above −2.5.

The American Society for Bone and Mineral Research (ASBMR) and Bone Health & Osteoporosis Foundation (BHOF) Task Force on Goal-Directed Osteoporosis Treatment suggests a minimum treatment T-score target of >−2.5 at the TH, FN, and LS.^1^ Higher treatment targets (> − 2.0) were recommended for individuals with a history of fracture or other major risk factors.^1^^,^^6^ The goal-directed approach suggests that initial treatment decisions, treatment sequences, and treatment changes be based on the likelihood of achieving and maintaining individualized treatment targets.

Abaloparatide is a selective activator of the parathyroid hormone receptor type 1 pathway that stimulates bone formation rapidly and prominently and later stimulates bone resorption to a lesser extent.^7^^,^^8^ In the ACTIVE trial, women were randomized to treatment with abaloparatide, teriparatide, or placebo. After 18 mo of abaloparatide treatment,^7^ mean increments with abaloparatide and teriparatide were 4.2% and 3.3% at the TH and 11.2% and 10.5% at the LS, respectively.^9^ The increments observed with 18 mo of abaloparatide during ACTIVE were extended after transition to alendronate for an additional 2 yr in the ACTIVExtend study.^10^ At 43 mo, mean BMD increased by 6.4% in the TH and 14.4% in the LS with abaloparatide followed by alendronate compared with placebo followed by alendronate (2.8% and 6.5%, respectively).^11^

The objective of this post hoc analysis of the ACTIVE and ACTIVExtend trials was to predict the probabilities of reaching target T-scores > − 2.5 at both the TH and LS over 12 and 18 mo of treatment with abaloparatide or teriparatide in women with baseline T-scores ≤ − 2.5 at the respective skeletal sites. These probabilities were also calculated in women who received 18 mo of abaloparatide or placebo, followed by 24 mo of treatment with alendronate in the ACTIVExtend trial.

## Methods

As previously described, the ACTIVE trial enrolled 2463 women aged 49 to 86 yr with osteoporosis, defined as prior vertebral or nonvertebral fracture and LS or FN T-score ≤−2.5 for age <65 yr or for women >65 yr of age, ≤−2.0 with prior fracture or ≤−3.0 without fracture.^8^ Women were randomized to receive daily subcutaneous abaloparatide 80 μg, teriparatide 20 μg, or placebo for 18 mo. At the conclusion of ACTIVE, 581 women from the placebo cohort and 558 women from the abaloparatide cohort were enrolled in the ACTIVExtend trial where they received alendronate (70 mg PO once weekly) for a further 24 mo.^10^

In this post hoc analysis, we determined the probability of achieving T-scores >−2.5 at 12 and 18 mo in women enrolled in the ACTIVE study who had baseline T-scores ≤−2.5 at the TH (*n* = 533) or LS (n = 1822). These probabilities were also determined in the ACTIVExtend study after a treatment sequence of abaloparatide or placebo for 18 mo followed by alendronate for 24 mo.

**Table 1:** Baseline characteristics of women with T-scores ≤−2.5 at TH or LS and available BMD data at 12 or 18 mo (from ACTIVE).

Variable	T-score ≤ −2.5 at the TH			T-score ≤ −2.5 at the LS		
	ABL (*N* = 145)	TPTD (*N* = 150)	PBO (*N* = 169)	ABL (*N* = 478)	TPTD (*N* = 505)	PBO (*N* = 527)
**Age, mean (SD), yr**	69.8 (6.9)	70.5 (6.9)	70.4 (6.7)	68.3 (6.7)	68.3 (6.3)	68.4 (6.4)
**BMI, mean (SD), kg/m^2^^a^**	23.1 (3.2)	23.3 (3.4)	23.1 (3.1)	24.5 (3.4)	24.6 (3.5)	24.7 (3.4)
**BMD T-score, mean (SD)**						
** LS**	−3.2 (0.9)	−3.1 (1.0)	−3.2 (0.9)	−3.3 (0.5)	−3.3 (0.5)	−3.3 (0.5)
** TH**	−2.9 (0.4)	−2.9 (0.4)	−2.9 (0.3)	−1.9 (0.7)	−1.9 (0.7)	−2.0 (0.8)
**≥1 Prevalent vertebral fracture(s), *n* (%)**	31/145 (21.4)	36/150 (24.0)	48/169 (28.4)	92/478 (19.2)	124/505 (24.6)	115/526 (21.9)
**≥1 Prior nonvertebral fracture(s), *n* (%)^b^**	37/145 (25.5)	35/150 (23.3)	47/169 (27.8)	122/478 (25.5)	123/505 (24.4)	141/527 (26.8)
**No history of prior fracture, *n* (%)**	57/145 (39.3)	70/150 (46.7)	72/169 (42.6)	211/478 (44.1)	228/505 (45.1)	234/527 (44.4)

Abbreviations: ABL, abaloparatide; PBO, placebo; TPTD, teriparatide.

a BMI is calculated as weight in kilograms divided by height in meters squared.

b Assessed within the last 5 yr based on fractures that occurred prior to visit 3 (day 1 of study). Excludes fractures of the spine, sternum, patella, toes, fingers, skull, and facial bones.

### Statistical analysis

Women with baseline TH or LS T-score ≤−2.5 and postbaseline BMD measurements at 12 or 18 mo were included. A logistic regression model was used to predict the probability of achieving a T-score >−2.5 at each respective site during 12 and 18 mo of treatment with abaloparatide or teriparatide. For the 43-mo measurements, women originally enrolled in the abaloparatide and placebo groups with a baseline TH or LS T-score ≤−2.5, and BMD measurements at both 18 and 43 mo. A logistic regression model was used to predict the probability of achieving a T-score >−2.5 at either TH or LS, respectively, during 3.5 yr of treatment with the sequence of abaloparatide/alendronate or placebo/alendronate. Baseline TH or LS T-scores within treatment groups were included in the corresponding logistic model.

## Results

In the ACTIVE population at baseline, mean TH and LS T-scores were −1.9 and −2.9, respectively; 23% of women enrolled had TH T-scores ≤−2.5 (*n* = 553) and 74% had LS T-scores ≤−2.5 (*n* = 1822). Average BMD levels and proportions of women with T-scores ≤−2.5 were balanced across the 3 treatment groups (Table 1). Mean age, BMI, TH, and LS T-scores, and prevalence of vertebral and nonvertebral fractures were also balanced across treatment groups. Of those with TH T-scores ≤−2.5, 32% had TH T-scores ≤−3.0 and of those with LS T-scores ≤−2.5 approximately 70% had an LS T-score ≤−3.0. These proportions were also balanced across groups. Descriptive characteristics for women included in the 12- or 18-mo analyses with T-scores ≤−2.5 are shown in Table 1.

For the 43-mo analyses, 260 and 852 women were included for the TH and LS probability estimates, respectively. At baseline, these women had characteristics very similar to those included in the 18-mo analyses and, again, the 2 groups were well balanced (Table 2).

**Table 2:** Baseline characteristics of the subgroup of women with T-scores ≤−2.5 at TH or LS and who had BMD data available at 43 mo (from ACTIVExtend).^a^

Variable	T-score ≤ −2.5 at the TH		T-score ≤ −2.5 at the LS	
	ABL/ALN (*N* = 110)	PBO/ALN (*N* = 123)	ABL/ALN (*N* = 373)	PBO/ALN (*N* = 395)
**Age, mean (SD), yr**	69.8 (6.2)	70.0 (6.1)	68.1 (6.5)	68.5 (6.2)
**BMI, mean (SD), kg/m^2^^b^**	23.1 (3.2)	22.84 (3.0)	24.5 (3.4)	24.6 (3.3)
**BMD T-score, mean (SD)**				
** LS**	−3.2 (0.9)	−3.2 (0.9)	−3.3 (0.5)	−3.3 (0.5)
** TH**	−2.9 (0.3)	−2.9 (0.3)	−1.9 (0.7)	−2.0 (0.7)
**≥1 Prevalent vertebral fracture(s), *n*/*N* (%)**	21/110 (19.1)	33/123 (2.8)	73/373 (19.6)	84/394 (21.3)
**≥1 Prior nonvertebral fracture(s), *n*/*N* (%)^c^**	27/110 (24.5)	29/123 (23.6)	89/373 (23.9)	98/395 (24.8)
**No history of prior fracture, *n*/*N* (%)**	48/110 (43.6)	57/123 (46.3)	169/373 (45.3)	185/395 (45.3)

Abbreviations: ABL, abaloparatide; ALN, alendronate; PBO, placebo; TPTD, teriparatide.

a Baseline was considered to be prior to randomization in ACTIVE.

b BMI is calculated as weight in kilograms divided by height in meters squared.

c Assessed within the last 5 yr based on fractures that occurred prior to visit 3 (day 1 of study). Excludes fractures of the spine, sternum, patella, toes, fingers, skull, and facial bones.

**Figure 1:**
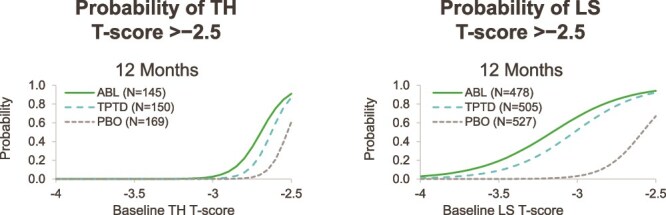
Probability of achieving T-score >−2.5 at TH and LS over 12 mo of treatment with abaloparatide, teriparatide, or placebo in the ACTIVE trial. Abbreviations: ABL, abaloparatide; PBO, placebo; TPTD, teriparatide.

As shown in Figure 1, over 12 mo of treatment in ACTIVE, at least 50% of women were likely to achieve TH T-scores >−2.5, with baseline T-scores of at least −2.7 for abaloparatide and −2.6 for teriparatide. For the LS, more than 50% of women were predicted to achieve an LS T-score >−2.5 with a baseline LS T-score as low as −3.2 on abaloparatide or −3.0 on teriparatide. In the placebo group, the probabilities of achieving TH or LS T-scores >−2.5 were very low.

**Figure 2:**
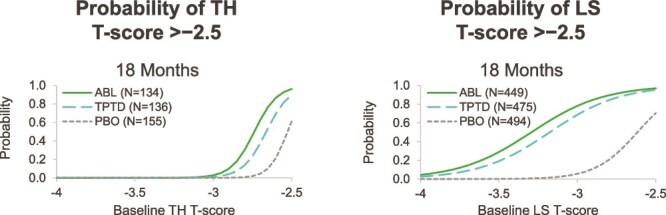
Probability of achieving T-score >−2.5 at TH and LS over 18 mo of treatment with abaloparatide, teriparatide, or placebo in the ACTIVE trial. Abbreviations: ABL, abaloparatide; PBO, placebo; TPTD, teriparatide.

As shown in Figure 2, with 18 mo of treatment in ACTIVE, in women with a baseline TH T-score ≥−2.7, more than 50% were likely to achieve TH T-scores >−2.5 with either abaloparatide or teriparatide. At least 50% of women who began with an LS T-score ≥−3.3, for those treated with abaloparatide, or ≥−3.2 for those treated with teriparatide, were predicted to achieve an LS T-score >−2.5.

**Figure 3:**
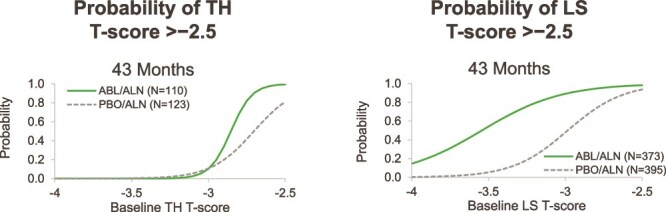
Probability of achieving T-scores >−2.5 at TH and LS over 43 mo of treatment with abaloparatide or placebo followed by alendronate in ACTIVExtend. Abbreviations: ABL, abaloparatide; ALN, alendronate; PBO, placebo.

Figure 3 illustrates the probabilities of achieving T-scores >−2.5 with 43 mo of sequential treatment (abaloparatide or placebo for 18 mo followed by alendronate for 24 mo) in the ACTIVExtend study. More than 50% of participants who began with a TH T-score as low as −2.9 were predicted to attain a T-score >−2.5 with abaloparatide/alendronate, and those beginning with a T-score as low as −2.7 in the placebo/alendronate group would reach a T-score >−2.5. More than 50% of women with baseline LS T-scores as low as −3.5 for those treated with the abaloparatide/alendronate sequence and −3.0 for those treated with the placebo/alendronate sequence were predicted to attain an LS T-score >−2.5.

## Discussion

In this post hoc analysis, we show that, after 18 mo of abaloparatide or teriparatide treatment in ACTIVE, women with starting TH T-scores as low as −2.7 for both or LS T-scores as low as −3.3 and −3.2, respectively, had a more than 50% likelihood of achieving T-scores >−2.5 at the respective sites. In the placebo group, which received calcium and vitamin D, probabilities of achieving TH T-scores or LS T-scores >−2.5 were very low. At month 43 with the abaloparatide/alendronate treatment sequence, more than 50% of women with baseline T-scores as low as −2.9 in the TH and −3.5 in the LS were likely to achieve a minimum target T-score >−2.5 at each site respectively, compared to −2.7 and −3.0 for placebo followed by alendronate.

Recently, the ASBMR/BHOF Task Force on Goal-Directed Osteoporosis Treatment published a position statement, providing an evidence-based summary of major clinical recommendations regarding treatment targets and strategies.^1^ Our findings help inform goal-directed treatment decisions based on the ASBMR/BHOF Task Force recommendations. The recommended goal-directed BMD target for patients with baseline T-scores <−2.5 at the TH, FN, and/or LS is at minimum a T-score >−2.5 at the respective skeletal site.^1^ Initial treatment selection should consider a ≥50% probability of attaining the T-score target over approximately 3 yr depending on the initial BMD (faster for patients with imminent fracture risk such as those with recent or multiple prior fractures). Achieving these T-score levels is associated with minimized risk of fracture in patients treated with various therapies, including osteoanabolic and antiresorptive treatments.^3–6^

Osteoanabolic agents reduce fracture risk more than antiresorptives and should be considered as initial therapy in patients at very high risk.^11^ However, use of osteoanabolic agents is usually limited to 1-2 yr. Consequently, sequenced treatment is required.^11^ As shown by our results, and in studies with romosozumab or teriparatide followed by denosumab or alendronate, initial osteoanabolic therapy followed by antiresorptive treatment shows rapid and greater BMD increases at the hip and spine than placebo followed by antiresorptive treatment or antiresorptive treatment alone.

Findings here for 2 yr of alendronate treatment are similar to those published previously from other analyses for 3 yr of alendronate treatment.^12^ With baseline T-scores below −2.7 in the TH and −3.0 in the LS, the probabilities of achieving T-scores >−2.5 were lower than 50% even after 3 yr of alendronate treatment. For patients who begin with T-scores below these levels, beginning with abaloparatide or teriparatide is much more likely to enable attainment of BMD treatment targets.^11^ This is consistent with studies of fracture reduction endpoints, indicating the superior antifracture efficacy of teriparatide vs risedronate^13^ and similar or even better antifracture efficacy of abaloparatide vs teriparatide.^8^ Our results for abaloparatide followed by alendronate over 3.5 yr are also similar to those seen with romosozumab followed by alendronate over 3 yr.^12^ With either regimen, at least 50% of women would likely achieve a T-score target >−2.5 beginning with a T-score as low as −2.9 in the TH or as low as −3.5 in the LS.

Limitations of this analysis include the lack of data for achievement of T-score targets with sequential treatment after teriparatide. Data here analyzed achievement of T-score targets after 3.5 yr of treatment, whereas other studies have analyzed data after 3 yr of treatment. Because of the study design, we do not have comparable probabilities for alendronate alone over 3.5 yr; however, BMD usually plateaus with alendronate over 2-3 yr^3^ and there were no apparent differences regarding the probabilities of attaining T-score targets with 2 vs 3 yr of alendronate therapy. Additionally, BMDs were significantly higher in the abaloparatide arm compared with placebo at baseline in the ACTIVExtend study,^10^ with BMD in the placebo group remaining near the ACTIVE baseline levels, which should be considered when interpreting the placebo/alendronate results. However, after 24 mo of alendronate, BMD increases from the ACTIVExtend baseline were similar in both the former abaloparatide and placebo groups. These results are based on trials of primarily white women and may not apply to other racial groups who have a lower risk of fracture. Because the abaloparatide study in men was small and only 1 yr long,^14^ we are unable to provide the same results for men and cannot assume that the findings will be the same as those seen in women. Because populations entering clinical trials vary, comparisons across trials should be undertaken cautiously. Even with the sequence of abaloparatide followed by alendronate, women who begin with TH T-scores ≤−3 have a low probability of achieving treatment targets; however, beginning with abaloparatide is likely to allow achievement of BMD levels closer to target than beginning with alendronate alone. Strengths of this study include the well-characterized BMD findings for the randomized treatment arms (abaloparatide, teriparatide, and placebo) over 18 mo and for the 2 treatment arms over the 43-mo treatment sequence (abaloparatide vs placebo followed by alendronate). The data from ACTIVE and ACTIVExtend are unique; there are no other similar studies that could be used to assess the reported probabilities.

## Conclusions

In conclusion, women with T-scores below −2.7 in the TH and −3.0 in the LS are not likely to attain minimum target T-scores >−2.5 with bisphosphonates only. In contrast, women who begin with T-scores above −2.9 in the TH or ≥−3.5 in the LS have a high probability of attaining target T-scores if given 3.5 yr of sequential treatment with abaloparatide followed by alendronate. In determining what treatment to initiate in patients at high or very high risk of fracture, baseline BMD and the probability of achieving T-score treatment targets should be considered.

## Data Availability

Data that underlie the results reported in a published article may be requested for further research 6 mo after completion of FDA or EMA regulatory review of a marketing application (if applicable) or 18 mo after trial completion (whichever is latest). Radius will review requests individually to determine whether (i) the requests are legitimate and relevant and meet sound scientific research principles, and (ii) are within the scope of the participants’ informed consent. Prior to making data available, requestors will be required to agree in writing to certain obligations, including without limitation, compliance with applicable privacy and other laws and regulations. Proposals should be directed to info@radiuspharm.com.
